# Candidate Genes and Molecular Markers Correlated to Physiological Traits for Heat Tolerance in Fine Fescue Cultivars

**DOI:** 10.3390/ijms19010116

**Published:** 2018-01-01

**Authors:** Yi Xu, Jinyu Wang, Stacy A. Bonos, William A. Meyer, Bingru Huang

**Affiliations:** Department of Plant Biology, Rutgers University, New Brunswick, NJ 08901, USA; xu@aesop.rutgers.edu (Y.X.); wangjinyu90@yahoo.com (J.W.); bonos@rutgers.edu (S.A.B.); wmeyer@aesop.rutgers.edu (W.A.M.)

**Keywords:** turfgrass, molecular marker, heat tolerance, leaf chlorophyll content, photosynthesis, membrane stability

## Abstract

Heat stress is one of the major abiotic factors limiting the growth of cool-season grass species during summer season. The objectives of this study were to assess genetic variations in the transcript levels of selected genes in fine fescue cultivars differing in heat tolerance, and to identify single nucleotide polymorphism (SNP) markers associated with candidate genes related to heat tolerance. Plants of 26 cultivars of five fine fescue species (*Festuca* spp.) were subjected to heat stress (38/33 °C, day/night temperature) in controlled environmental growth chambers. Physiological analysis including leaf chlorophyll content, photochemical efficiency, and electrolyte leakage demonstrated significant genetic variations in heat tolerance among fine fescue cultivars. The transcript levels of selected genes involved in photosynthesis (RuBisCO activase, Photosystem II CP47 reaction center protein), carbohydrate metabolism (Sucrose synthase), energy production (ATP synthase), growth regulation (Actin), oxidative response (Catalase), and stress protection (Heat shock protein 90) were positively correlated with the physiological traits for heat tolerance. SNP markers for those candidate genes exhibited heterozygosity, which could also separate heat-sensitive and heat-tolerant cultivars into clusters. The development of SNP markers for candidate genes in heat tolerance may allow marker-assisted breeding for the development of new heat-tolerant cultivars in fine fescue and other cool-season grass species.

## 1. Introduction

Heat stress is one of the primary abiotic factors limiting growth of cool-season species. Plant tolerance to heat stress involves various aspects at the physiological, biochemical, and molecular levels, including maintenance of active cell growth, photosynthesis, and activation of stress protection systems (for review, see [1]). Maintenance of active photosynthesis, energy and sugar metabolism, as well as cell membrane stability has been established to correlate with improved heat tolerance in various plant species, including perennial grass species [2,3,4,5,6,7]. Stress-protective proteins, such as heat shock proteins and antioxidant enzymes are also highly corrected to heat tolerance [3,8,9]. Linking physiological traits to candidate gene expression and association with molecular makers is an effective approach to developing stress-tolerant cultivars based on desirable traits of plant species [10,11].

Numerous genes responsive to heat stress have been found in various plant species using genomic, transcriptomic, and proteomic analysis [12,13,14,15,16,17,18]. Many genes are known to play important roles in heat tolerance [12,14,19,20], including those involved in photosynthesis (i.e., Ribulose-1,5-biphosphate carboxylase/oxygenase (RuBisCO) large subunit (RBCL), RuBisCO small subunit (RBCS), RuBisCO activase beta subunit (RCAB), Photosystem II CP47 reaction center protein (CP47), Chlorophyll a/b binding protein (CAB), Ferredoxin-NADP reductase (FNR)), energy metabolism (ATP synthase alpha subunit (ATPA), ATPase (ATPASE), Glyceraldehyde 3-phosphate dehydrogenase (GAPDH) beta subunit (GAPDH)), stress protection (Heat shock protein 26 (HSP26), HSP70, HSP90 catalase alpha subunit (CATA)). Candidate genes with known biological functions associated with physiological traits have been found to be particularly useful for genetic improvement in stress tolerance through either genetic modification or molecular breeding in both annual crops and perennial grasses [21,22,23,24,25]. Single nucleotide polymorphism (SNP) marker is one of the most widely-used DNA markers in marker-assisted selection, which is a very attractive and potential genetic marker for genetic breeding [26]. The associations of SNP and physiological trait have also been extensively studied [27,28]. However, the association of candidate genes with genetic variations for heat tolerance and markers linked to physiological traits are not well documented for perennial grass species.

Fine fescue is a cool-season perennial grass species, which has relatively low-maintenance characteristics in turf situations [29]. Fine fescue has good turf quality in spring and fall, but may experience significant summer dormancy in full-sun locations during summer [30]. Previous studies showed that heat stress caused differential profiles of ethyl sterols and fatty acids in leaf cell membranes between heat-tolerant and heat-sensitive fine fescue cultivars [31]. Several membrane proteins related to photosynthesis, electron transport, signaling, stress defense, and protein modification were also reported to be more upregulated or less downregulated in heat-tolerant cultivar than in heat-sensitive cultivar [31]. The genetic variability of heat tolerance in fine fescue makes it of great value for the identification of candidate genes and DNA markers associated with heat tolerance in cool-season perennial grass species. The objectives of this study were to assess the genetic variation in the transcript levels of several genes related to physiological performance of fine fescue cultivars showing contrasting heat tolerances, and to identify single nucleotide polymorphism (SNP) markers associated with such candidate genes related to heat tolerance.

## 2. Results

### 2.1. Genetic Variations in Physiological Traits Associated with Heat Tolerance

**Figure 1:**
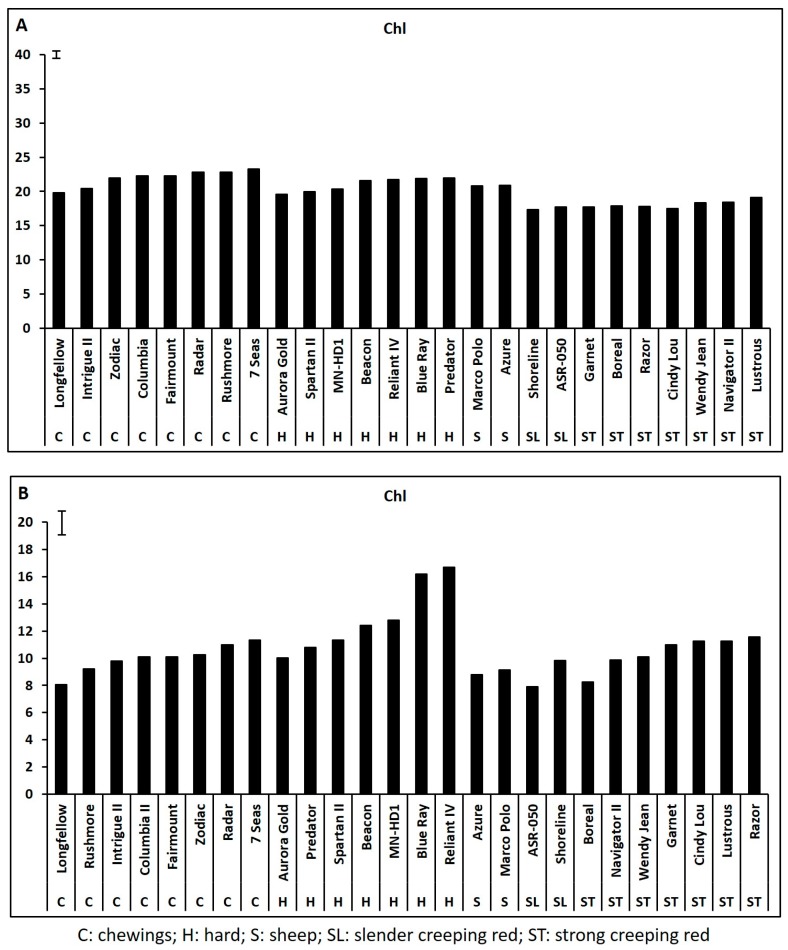
Leaf chlorophyll (Chl) content (μmol g^−1^ plant dry weight) in fine fescue cultivars under control condition (**A**) and on 35 days of heat stress (**B**). Bar represents least significant difference (LSD) at α < 0.05 (*n* = 4).

**Figure 2:**
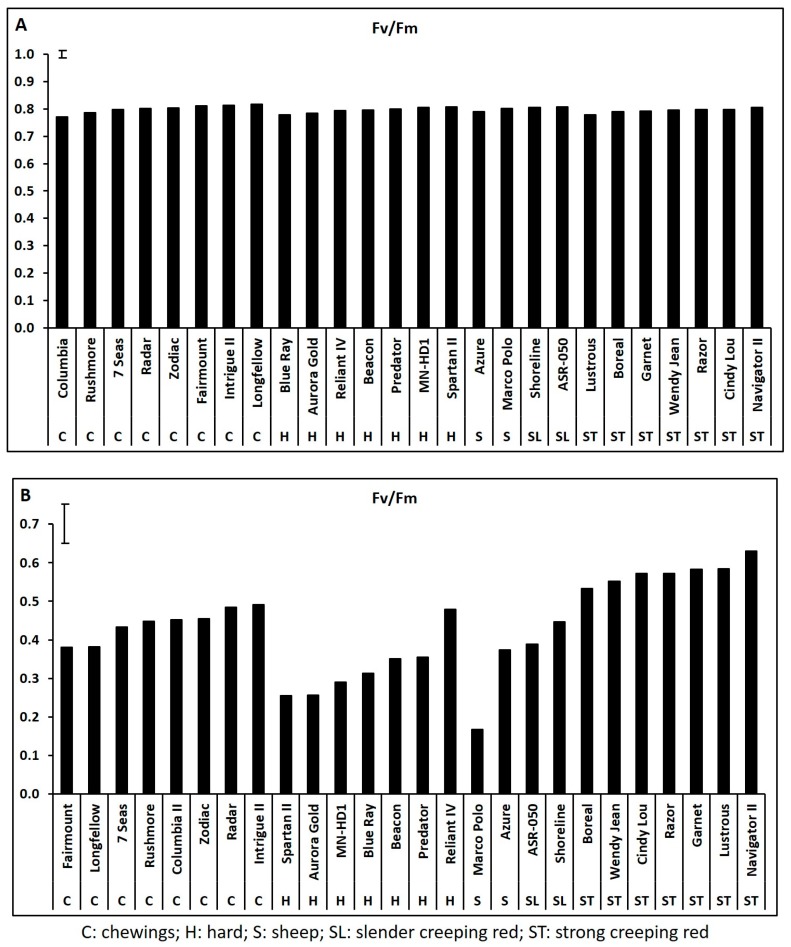
Leaf photochemical efficiency (Fv/Fm ratio) in fine fescue cultivars under control condition (**A**) and on 35 days of heat stress (**B**). Bar represents least significant difference (LSD) at α < 0.05 (*n* = 4).

**Figure 3:**
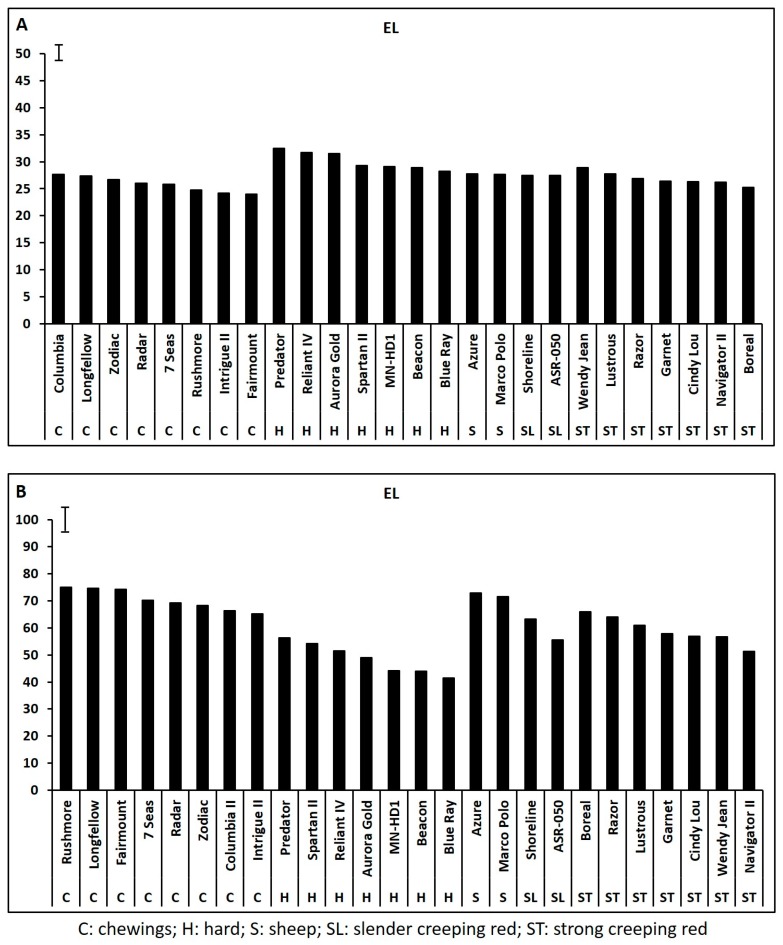
Leaf electrolyte leakage (EL, %) in fine fescue cultivars under control condition (**A**) and on 35 days of heat stress (**B**). Bar represents least significant difference (LSD) at α < 0.05 (*n* = 4).

Leaf chlorophyll content and photochemical efficiency declined significantly while electrolyte leakage increased significantly during 35 days of heat stress for all 26 cultivars based on the data collected weekly at 7, 14, 21, 28, and 35 days of heat stress (data not shown), and the adverse effects of heat stress and the genetic variations in all physiological parameters were most pronounced at 35 days of heat stress. Cultivars within each species exhibited significant variations in Chl content, Fv/Fm, and EL at 35 days of heat stress while none of the physiological traits differed among cultivars within each species under non-stress control conditions (Figure 1, Figure 2 and Figure 3).

**Figure 4:**
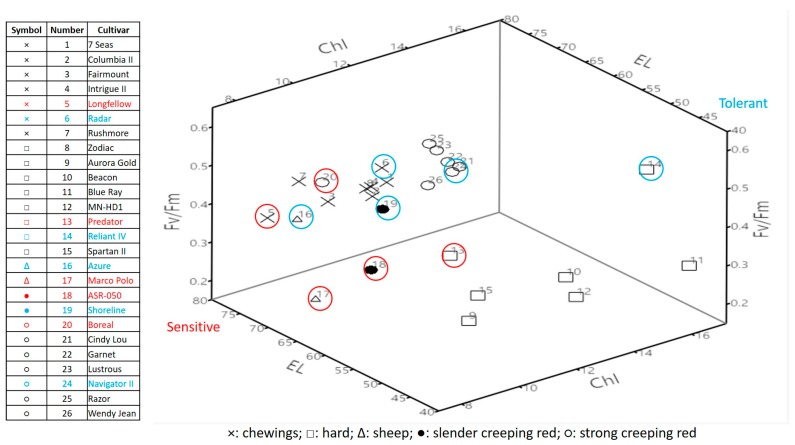
3D scatter plot using leaf chlorophyll (Chl) content, photochemical efficiency (Fv/Fm) and electrolyte leakage (EL) in fine fescue cultivars on 35 days of heat stress.

The combined analysis of all three physiological traits (Figure 4) showed that the most heat-sensitive and most heat-tolerant cultivars for each fine fescue species were as follows: ‘Longfellow’ and ‘Radar’ for chewings fescue, ‘Predator’ and ‘Reliant IV’ for hard fescue, ‘Marco Polo’ and ‘Azure’ for sheep’s fescue, ‘ASR-050’ and ‘Shoreline’ for slender creeping red fescue, and ‘Boreal’ and ‘Navigator II’ for strong creeping red fescue, and there were greater cultivar variations or separation in heat tolerance for hard fescue and chewings fescue than other species. Therefore, two cultivars contrasting in heat tolerance, heat-tolerant ‘Longfellow’ and heat-sensitive ‘Radar’ for chewings fescue, heat-sensitive ‘Predator’ and heat-tolerant ‘Reliant IV’ for hard fescue, were further examined for candidate genes associated with the differential heat tolerance.

### 2.2. Differential Expression of Candidate Genes in Heat-Sensitive and Heat-Tolerant Cultivars

A total of 16 genes were selected for transcript level analysis using qRT-PCR (Table 1), including those with functions in photosynthesis [Ribulose-1,5-biphosphate carboxylase/oxygenase (RuBisCO) large subunit (RBCL), RuBisCO small subunit (RBCS), RuBisCO activase beta subunit (RCAB), Photosystem II CP47 reaction center protein (CP47), Chlorophyll a/b binding protein (CAB), Ferredoxin-NADP reductase (FNR)], energy metabolism [ATP synthase alpha subunit (ATPA), ATPase (ATPASE), Glyceraldehyde 3-phosphate dehydrogenase beta subunit (GAPDH)], sugar metabolism [Sucrose synthase 1 (SS1)], growth regulation [Actin (ACTIN), Tubulin beta subunit (TUBB)], stress protection [Heat shock protein 26 (HSP26), HSP70, HSP90, Catalase alpha subunit (CATA)].

**Figure 5:**
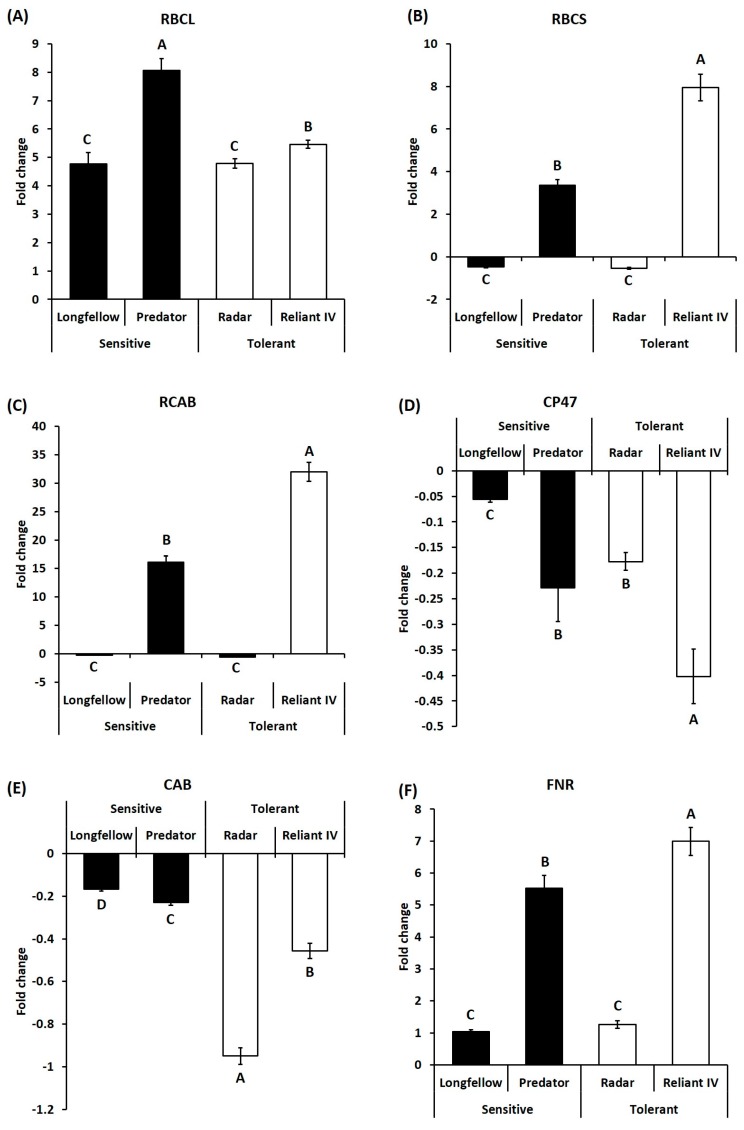
Relative expressions of genes related to photosynthesis at 35 d of heat stress, represented by fold change of transcript level under heat stress compared with that under control condition. Bar on each column represents standard error of four biological replicates (*n* = 4). Different letters indicate significant differences at the *p* level of 0.05. (**A**) RBCL: Rubisco large subunit; (**B**) RBCS: Rubisco small subunit; (**C**) RCAB: Rubisco activase beta subunit; (**D**) CP47: Photosystem II CP47 reaction center protein; (**E**) CAB: Chlorophyll a/b binding protein; (**F**) FNR: Ferredoxin-NADP reductase.

For genes involved in photosynthesis, transcript levels of RBCL were significantly upregulated at 35 d of heat stress in both heat-sensitive cultivars (by 4.76- and 8.06-fold in ‘Longfellow’, ‘Predator’) and heat-tolerant cultivars (by 4.78- and 5.47-fold in ‘Radar’ and ‘Reliant IV’) of chewings fescue and hard fescue (Figure 5A). Transcript levels of RBCS were downregulated in heat-sensitive cultivar (by 0.48-fold in ‘Longfellow’) and heat-tolerant cultivar (by 0.54-fold in ‘Radar’) of chewings fescue, but upregulated in the heat-sensitive ‘Predator’ (3.35-fold) and heat-tolerant ‘Reliant IV’ (7.95-fold) of hard fescue (Figure 5B). The transcript levels of RCAB were significantly upregulated in heat-sensitive cultivar (16.1-fold in ‘Predator’) and heat-tolerant cultivar (31.9-fold in ‘Reliant IV’) in hard fescue, but significantly downregulated in a heat-sensitive cultivar (0.26-fold in ‘Longfellow’) and a heat-tolerant cultivar (0.56-fold in ‘Radar’) of chewings fescue (Figure 5C). The transcript levels of CP47 were significantly downregulated in a heat-sensitive cultivar (0.06- and 0.23-fold in ‘Longfellow’ and ‘Predator’) and in heat-tolerant cultivars (0.18- and 0.40-fold in ‘Radar’ and ‘Reliant IV’) of both chewings fescue and hard fescue (Figure 5D). Heat stress caused significant downregulation of CAB transcripts in heat-sensitive cultivars (0.17- and 0.23-fold in ‘Longfellow’, ‘Predator’) and heat-tolerant cultivars (0.95- and 0.46-fold in ‘Radar’ and ‘Reliant IV’) (Figure 5E). The transcript levels of FNR were significantly upregulated by heat stress in heat-sensitive cultivars (1.04- and 5.52-fold in ‘Longfellow’ and ‘Predator’) and heat-tolerant cultivars (1.22- and 6.99-fold in ‘Radar’ and ‘Reliant IV’) (Figure 5F).

**Figure 6:**
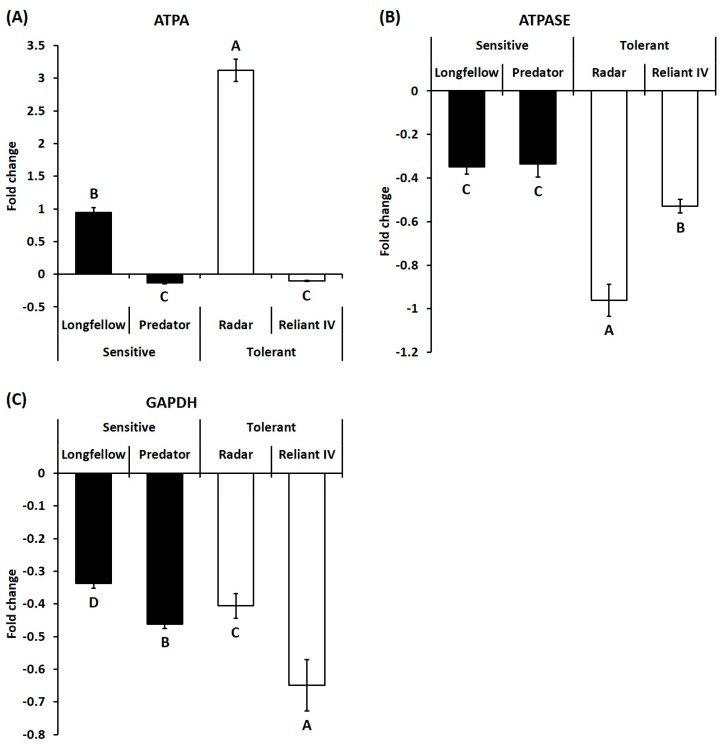
Relative expressions of genes related to energy production at 35 days of heat stress, represented by fold change of transcript level under heat stress compared with that under control condition. Bar on each column represents standard error of four biological replicates (*n* = 4). Different letters indicate significant differences at the *p* level of 0.05. (**A**) ATPA: ATP synthase alpha subunit; (**B**) ATPASE: ATPase; (**C**) GAPDH: Glyceraldehyde 3-phosphate dehydrogenase.

For genes involved in energy metabolism, heat stress caused significant downregulation of ATPA transcript levels in heat-sensitive cultivars (0.95- and 0.13-fold in ‘Longfellow’ and ‘Predator’) of chewings fescue and hard fescue and heat-tolerant cultivar of hard fescue (0.10-fold in ‘Reliant IV’), but significantly upregulated in heat-tolerant cultivar (3.12-fold in ‘Radar’) of chewings fescue (Figure 6A). The transcript levels of ATPASE were significantly downregulated in heat-sensitive cultivars (0.35- and 0.34-fold in ‘Longfellow’ and ‘Predator’) and heat-tolerant cultivars (0.96- and 0.53-fold in ‘Radar’ and ‘Reliant IV’) of both fine fescue species (Figure 6B). Transcript levels of GAPDH were downregulated in heat-sensitive cultivars (0.34- and 0.46-fold in ‘Longfellow’ and ‘Predator’) and heat-tolerant cultivars (0.41- and 0.65-fold in ‘Radar’ and ‘Reliant IV’) of both species (Figure 6C).

**Figure 7:**
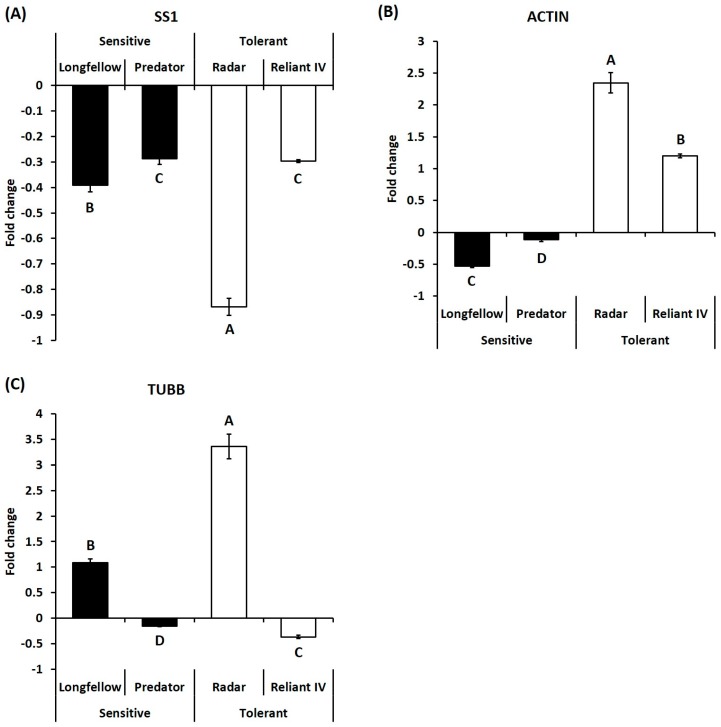
Relative expressions of genes related to sugar metabolism and growth at 35 days of heat stress, represented by fold change of transcript level under heat stress compared with that under control condition. Bar on each column represents standard error of four biological replicates (*n* = 4). Different letters indicate significant differences at the *p* level of 0.05. (**A**) SS1: Sucrose synthase 1; (**B**) ACTIN: Actin; (**C**) TUBB: Tubulin beta subunit.

For genes in sugar metabolism, transcript levels of SS1 were significantly downregulated under heat stress in heat-sensitive cultivars (0.39- and 0.29-fold in ‘Longfellow’ and ‘Predator’) and heat-tolerant cultivars (0.87- and 0.30-fold in ‘Radar’ and ‘Reliant IV’) (Figure 7A). In growth regulation, transcript levels of ACTIN were significantly downregulated under heat stress in heat-sensitive cultivars (0.53- and 0.12-fold in ‘Longfellow’ and ‘Predator’), but significantly upregulated in heat-tolerant cultivars (2.37- and 1.20-fold in ‘Radar’ and ‘Reliant IV’) (Figure 7B). The transcript levels of TUBB were significantly upregulated in a heat-sensitive cultivar (1.09-fold in ‘Longfellow’) and a heat-tolerant cultivar (3.36-fold in ‘Radar’) of chewings fescue, but significantly downregulated in heat-sensitive ‘Predator’ (0.16-fold) and heat-tolerant ‘Reliant IV’ (0.36-fold) of hard fescue (Figure 7C).

**Figure 8:**
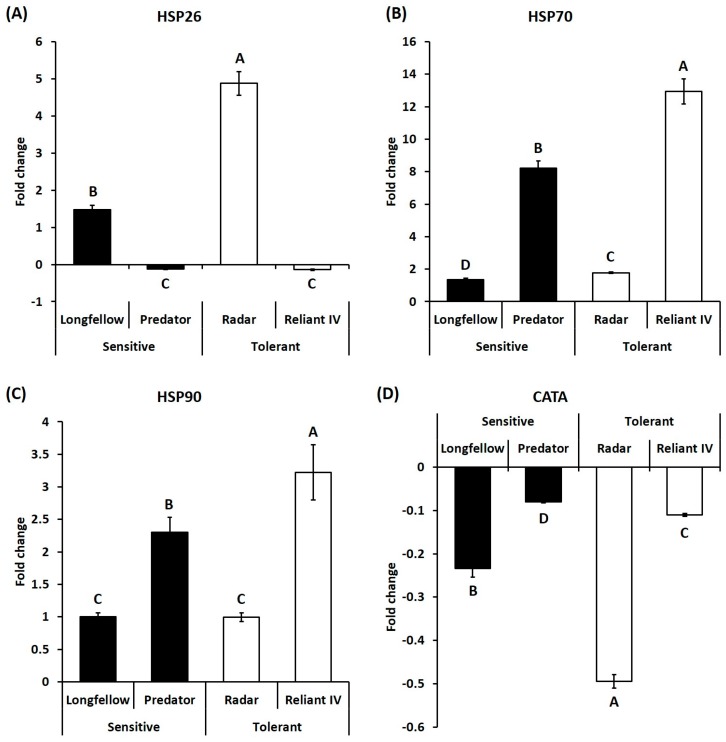
Relative expressions of genes related to stress protection at 35 days of heat stress, represented by fold change of transcript level under heat stress compared that under control condition. Bar on each column represents standard error of four biological replicates (*n* = 4). Different letters indicate significant differences at the *p* level of 0.05. (**A**) HSP26: Heat shock protein 26; (**B**) HSP70: Heat shock protein 70; (**C**) HSP90: Heat shock protein 90; (**D**) CATA: Catalase alpha subunit.

For stress protection, heat stress caused differential expression patterns of HSP26 in different cultivars, including significant upregulation in heat-sensitive cultivars (1.47-fold in ‘Longfellow’) and heat-tolerant cultivar (4.88-fold in ‘Radar’) of chewings fescue, and significant downregulation in heat-sensitive cultivar (0.13-fold in ‘Predator’) and heat-tolerant ‘Reliant IV’ (0.14-fold) of hard fescue (Figure 8A). Heat stress also led to significant upregulation of HSP70 transcript levels in heat-sensitive cultivars (1.35- and 8.21-fold in ‘Longfellow’ and ‘Predator’) and heat-tolerant cultivars (1.78- and 12.9-fold in ‘Radar’ and ‘Reliant IV’) (Figure 8B). Heat stress also resulted in significant upregulation of HSP90 transcript levels in a heat-sensitive cultivar (2.30-fold in ‘Predator’) and a heat-tolerant cultivar (3.22-fold in ‘Reliant IV’), but no significant effects were seen in the heat-sensitive cultivar ‘Longfellow’ and heat-tolerant ‘Radar’ of chewings fescue (Figure 8C). The transcript levels of CATA were significantly downregulated by heat stress in heat-sensitive cultivars (0.23- and 0.08-fold in ‘Longfellow’ and ‘Predator’) and heat-tolerant cultivars (0.50- and 0.11-fold in ‘Radar’ and ‘Reliant IV’) of both species (Figure 8D).

### 2.3. Identification of SNPs in Candidate Gene Sequences and Correlations with Heat Tolerance

To identify candidate gene-based SNP markers correlated to the genetic variations in heat tolerance of fine fescue species, SNPs for seven candidate genes (ACTIN, ATPA, CATA, CP47, HSP90, RCAB, and SS1) in different functional categories that exhibited significant differential responses to heat stress between heat-tolerant and heat-sensitive cultivars for chewings fescue and hard fescue, were compared among the most heat-sensitive and most heat-tolerant cultivars of five fine fescue species for all three physiological traits (Chl content, Fv/Fm, and EL).

**Figure 9:**
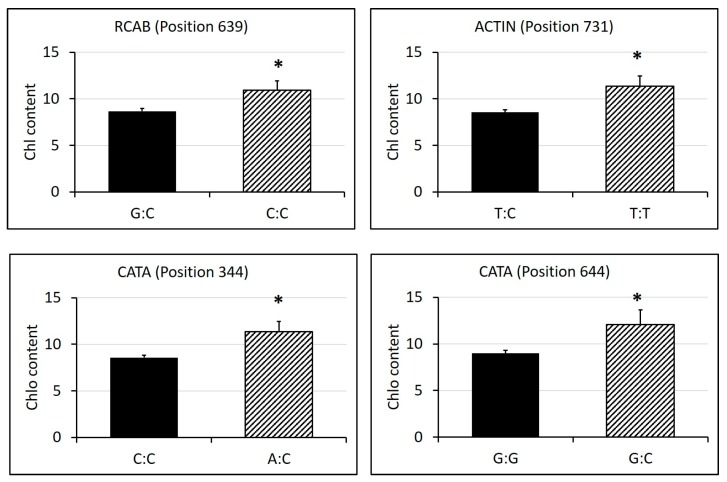
SNPs in candidate genes that showed differential leaf chlorophyll (Chl) content levels between homozygous and heterozygous alleles under 35 days of heat stress. SNP genotypes and corresponding numbers of cultivars are labelled in the x-axis. Bars represent standard errors of each corresponding numbers of biological replicates (cultivars), and asterisks represent significant differences at *p* level of 0.05.

**Figure 10:**
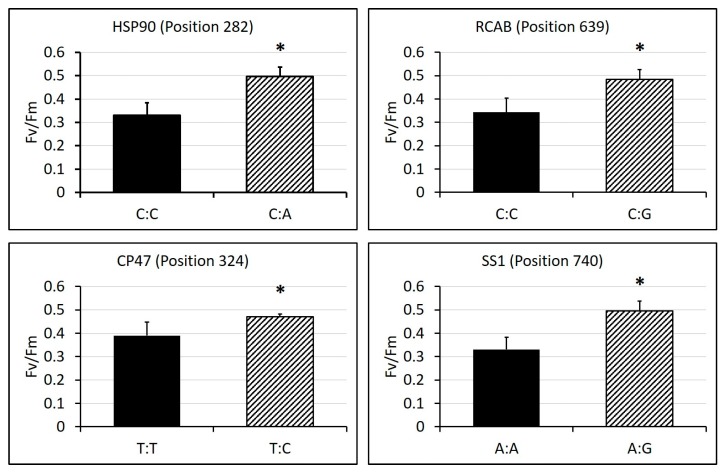
SNPs in candidate genes that showed differential photochemical efficiency (Fv/Fm) levels between homozygous and heterozygous alleles under 35 days of heat stress. SNP genotypes and corresponding numbers of cultivars are labelled in the x-axis. Bars represent standard errors of each corresponding numbers of biological replicates (cultivars), and asterisks represent significant differences at *p* level of 0.05.

**Figure 11:**
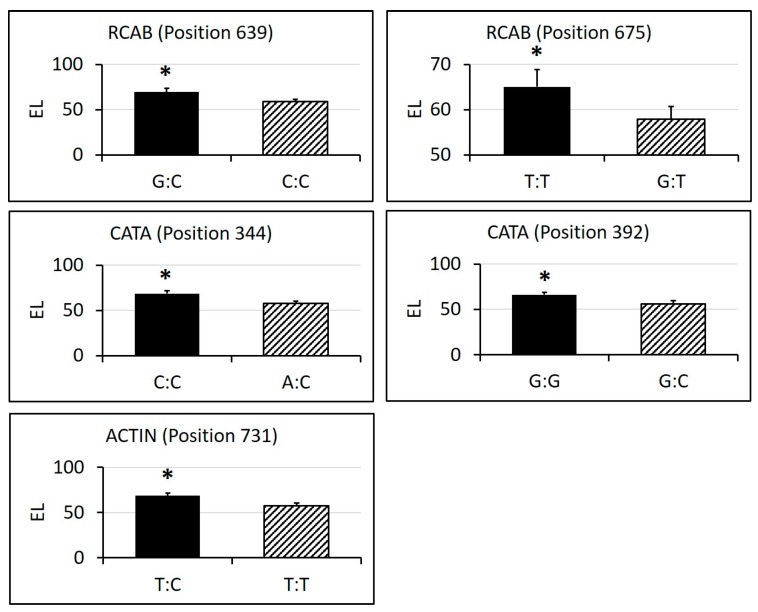
SNPs in candidate genes that showed differential electrolyte leakage (EL) levels between homozygous and heterozygous alleles under 35 days of heat stress. SNP genotypes and corresponding numbers of cultivars are labelled in the x-axis. Bars represent standard errors of each corresponding numbers of biological replicates (cultivars), and asterisks represent significant differences at *p* level of 0.05.

The sequencing alignment revealed a different number of SNPs in these genes, ranging from nine to 58 (Table 2, Tables S1 and S2). Among the SNPs found, 13 of them showed significant differences in physiological traits between homozygous alleles and heterozygous alleles. Cultivars having heterozygous alleles of SNPs in position 344 and 644 in CATA showed significantly higher chlorophyll content than those having homozygous alleles, and cultivars having homozygous alleles of SNPs in position 639 of RCAB and position 731 of ACTIN showed significantly higher chlorophyll content than those having heterozygous alleles (Figure 9). For photochemical efficiency, cultivars having heterozygous alleles of SNPs in position 282 of HSP90, position 639 of RCAB, position 324 of CP47 and position 740 of SS1 showed significantly higher Fv/Fm than those having homozygous alleles (Figure 10). For electrolyte leakage, cultivars having heterozygous alleles of SNPs in position 675 of RCAB, position 344 and 392 of CATA showed significantly lower EL than those having homozygous alleles, while cultivars having homozygous alleles of SNPs in position 639 of RCAB and position 731 of ACTIN showed significantly lower EL than those having heterozygous alleles (Figure 11). In addition, there was no amino acid substitution found in any of the heterozygous alleles of these SNPs, compared with their homozygous alleles, respectively.

**Figure 12:**
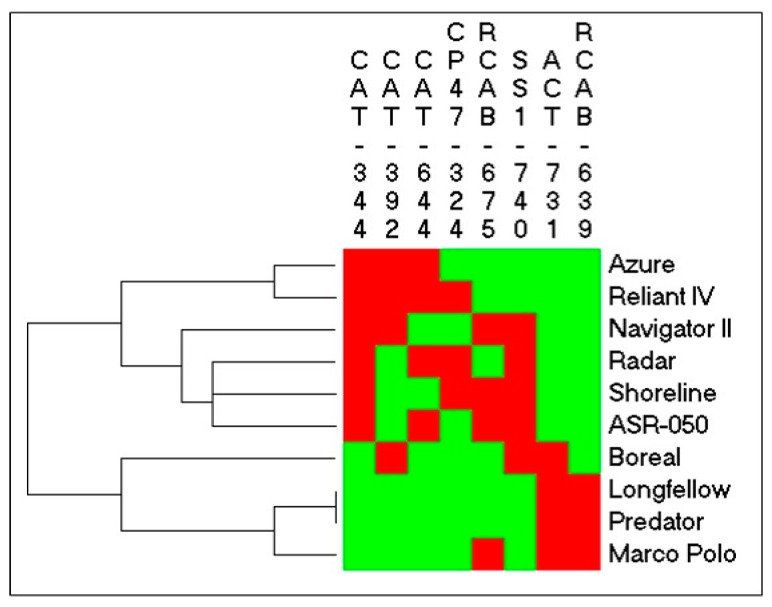
Two-way clustering analysis of SNPs in fine fescue cultivars. Green and red indicate a homozygous and heterozygous allele in each SNP position, respectively. The column name is the candidate gene and SNP position, and the row name is the cultivar. The Pearson’s R coefficient for the rows (cultivars) was 0.70, and that for the columns (SNPs) was 0.71.

Two-way clustering analysis was performed using all the above SNPs, based on their heterozygosity for each cultivar (Figure 12). The Pearson’s R coefficient for the rows (cultivars) was 0.70, and that for the columns (SNPs) was 0.71. It was shown that all 10 cultivars could be separated into two groups based on the SNPs, with all heat-tolerant cultivars (‘Azure’ of sheep’s fescue, ‘Reliant IV’ of hard fescue, ‘Navigator II’ of strong creeping red fescue, ‘Radar’ of chewings fescue, ‘Shoreline’ of slender creeping red fescue) and the heat-sensitive ‘ASR-050’ of slender creeping red fescue in the heat-tolerant class, while the other heat-sensitive cultivars, ‘Boreal’ in strong creeping red fescue, ‘Longfellow’ in chewings fescue, ‘Predator’ in hard fescue, and ‘Marco Polo’ in sheep’s fescue, grouped in the heat tolerance class.

## 3. Materials and Methods

### 3.1. Plant Materials and Growth Conditions

Plants of 26 cultivars from five fine fescue species were collected from the turfgrass research farm at Rutgers University, including eight chewings fescue (*Festuca rubra* subsp. commutata) cultivars (Zodiac, Intrigue II, Radar, Fairmount, Rushmore, 7 Seas, Columbia II, Longfellow), seven hard fescue (*F. brevipila*) cultivars (Blue Ray, Beacon, Spartan II, MN-HD1, Aurora Gold, Predator, Reliant IV), two sheep’s fescue (*F. ovina*) cultivars (Azure, Marco Polo), and nine strong and slender creeping red fescue (*F. rubra*) cultivars (Navigator II, Boreal, Lustrous, Garnet, Wendy Jean, Razor, Cindy Lou, Shoreline, ASR-050). Plants were transplanted into plastic pots (15 cm in diameter and 20 cm deep) filled with a 1:1 (*w*/*w*) mixture of soil and peat moss, and established in a greenhouse for 6 weeks, with an average of day/night temperature at 23/20 °C, and about 720 μmol m^−2^ s^−1^ photosynthetically active radiation (PAR) from sunlight and supplemental lighting. Plants were irrigated every day, trimmed twice every week to maintain 7 cm canopy height, and fertilized every 4 days with equal amount of half-strength Hoagland’s nutrient solution [32]. Plants were then moved to growth chambers (Environmental Growth Chambers, Chagrin Falls, OH, USA) for different temperature treatments. The growth chamber conditions were controlled as follows: 14 h photoperiod, 680 μmol m^−2^ s^−1^ PAR, 50% relative humidity, 22/18 °C (day/night) during plant establishment.

### 3.2. Temperature Treatments

For heat stress, plants were exposed to 38/33 °C (day/night) and for control treatment, plants were maintained at 22/18 °C (day/night), with other environmental conditions being the same for both temperature treatments. Each temperature treatment was repeated in four growth chambers. Each cultivar had four replicates (pots) placed randomly within each of four chambers for each temperature treatment. Plants were watered daily and fertilized weekly with equal amount of half-strength Hoagland’s nutrient solution. The experiment design was a split-plot on randomized complete block design (RCBD), with chambers as blocks, temperatures as main plots and cultivars as sub-plots.

### 3.3. Physiological Analysis

Chlorophyll (Chl) content was measured according to the protocol of Hiscox and Israelstom [33] with minor modifications. Approximately 0.1 g fresh leaf was extracted in 10 mL of dimethyl sulfoxide for 5 days. The extract solution was measured in a spectrophotometer (Spectronoic Instruments, Rochester, NY, USA) at 663 and 645 nm. The leaf tissue was dried in an 80 °C oven for 3 days and weighed to obtain dry weight. The Chl content was calculated on a dry weight basis using the equations described by Arnon [34].

Photochemical efficiency (Fv/Fm) was estimated by measuring chlorophyll fluorescence using a fluorescence induction monitor (Fim 1500, Dynamax, Houston, TX, USA). Leaves were first adapted in dark for 30 min, and photochemical efficiency was calculated by the ratio of variable fluorescence (Fv) to maximal fluorescence (Fm).

Leaf membrane stability was estimated via electrolyte leakage (EL) according to the method of Blum and Ebercon [35] with slight modifications. About 0.1 g fresh leaf was placed in a 50 mL tube containing 35 mL pure water and agitated for 16 h. The initial conductance reading (C_i_) of incubated solution was taken using a conductivity meter (YSI Inc., Yellow Springs, OH, USA). Tubes were then autoclaved at 120 °C for 20 min to kill leaf tissue, and agitated for an additional 16 h after which time a final conductance reading (C_max_) was measured. Electrolyte leakage was calculated as C_i_/C_max_ × 100%.

### 3.4. Gene Expression Analysis

Candidate genes were selected based on either our previous research [16,17] or searching against NCBI EST database using gene sequences from model plant species (*Arabidopsis thaliana*, *Oryza sativa*, or *Brachypodium distachyon*). Candidate gene expression was measured using quantitative reverse transcription polymerase chain reaction (qRT-PCR). Plant leaves were harvested at 0 and 35 d of heat stress and frozen in liquid nitrogen for later use. RNA was extracted using TRIzol reagent (Life Technologies, Carlsbad, CA, USA), and treated with TURBO DNase kit (Life Technologies). RNA quality and quantity were assessed using NanoDrop 1000 spectrophotometer (Thermo Fisher Scientific, Waltham, MA, USA). Equal amount (2 μg) of RNA was used to generate cDNA with a high-capacity cDNA synthesis kit (Life Technologies). The synthesized cDNA was amplified in a StepOnePlus Real-Time PCR system (Life Technologies) using the following parameters: pre-heat cycle of 95 °C for 3 min, 40 cycles of 95 °C denaturation for 30 s per cycle, and 60 °C annealing/extension for 30 s. Power SYBR Green PCR Master Mix (Life Technologies) was the intercalating dye used to detect gene expression level. Primers were designed using PrimerQuest tool (Integrated DNA Technologies, Coralville, IA, USA). Gene name, accession number, forward and reverse primer sequences are provided in Table 1. A melting curve analysis was performed for each primer set to confirm its specificity. Elongation factor 1 alpha (EF1A) was used as the reference gene, since its expression was consistent throughout cultivars and treatments. A ΔΔCt method was used to calculate the relative expression level between genes of interest and reference gene, respectively [36]. Four biological replicates (*n* = 4) were performed for each gene.

### 3.5. SNP Identification

Primers used for amplifying candidate gene sequences were listed in Table 3. PCR reaction was conducted by using Phusion Flash high-fidelity PCR master mix (Thermo Fisher Scientific). PCR product was first visualized in 1% agarose gel under UV light, recovered using GeneJET gel extraction and DNA cleanup micro kit (Thermo Fisher Scientific), and used for Sanger sequencing (GenScript, Piscataway, NJ, USA).

SNPs were identified using NovoSNP program [37], which could detect heterozygous sequence data based on a cumulative scoring scheme. Multiple alignments were performed by default value, and SNP positions and the heterozygous alleles were detected and manually filtered with threshold of FScore = 10. SNPs in each candidate gene were then grouped based on three physiological parameters (Chl content, Fv/Fm, or EL), respectively.

Two-way clustering analysis was performed using PermutMatrix 1.9.3 [38], with the following setting: Clustering method: McQuitty’s criteria; Tree seriation rule: Multiple-fragment heuristic; Dissimilarity: Euclidean distance.

### 3.6. Statistical Analysis

Effects of temperature treatments, cultivar variations, and temperature and cultivar interactions were analyzed using the general linear procedure for analysis of variance (ANOVA) in SAS v9.2 (SAS Institute, Cary, NC, USA). The significance of the mean differences was separated by Fisher’s protected LSD (α < 0.05) or Student’s *t*-test at the *p* level of < 0.05.

## 4. Discussion

Leaf chlorophyll content, photochemical efficiency, and electrolyte leakage are commonly-used, reliable parameters for the evaluation of genetic variations in heat tolerance, as they are key physiological traits affecting photosynthesis [39,40,41]. Photosynthesis, energy metabolism and sugar metabolism are primary physiological processes controlling plant stress adaptation [42]. In addition, metabolic factors and genes involved in stress defense, such as antioxidant defense and heat shock protection also play critical roles in plant tolerance to heat stress, as discussed in the introduction. To our knowledge, this is the first study correlating physiological traits to candidate genes and molecular markers for fine fescue species and cultivars with genetic variations for heat tolerance. The relationships between transcript levels, SNP-based markers, and the mechanisms of heat tolerance in fine fescue cultivars, relating to photosynthesis, sucrose synthesis, antioxidant system, stress protection, and growth regulation, are discussed as follows.

Plant photosynthesis is one of the most sensitive biological processes to heat stress [43]. Among many of the components involved in photosynthesis, RuBisCO activase is one of the most important enzymes regulating the activity of RuBisCO in the Calvin cycle, which also plays a pivotal role in plant response to high temperature [44]. In our study, the transcript levels of RuBisCO activase beta (RCAB) were more upregulated in heat-tolerant cultivar (‘Reliant IV’) than heat-sensitive cultivar (‘Predator’) of hard fescue. In addition, cultivars with homozygous alleles of SNP at position 639 of RCAB showed significantly higher chlorophyll content and photochemical efficiency, while cultivars with homozygous alleles of SNP at position 639, but heterozygous alleles of SNP at position 675 of RCAB showed significantly lower EL. It is thus clear that heat stress caused differential declines of leaf chlorophyll content and photochemical efficiency in fine fescue cultivars, while the transcript levels and SNP genotypes in RCAB could correlate with such declines. The relationship between RCAB and EL needs to be elucidated. All of the above observations pointed out that RCAB is a good candidate gene to select for genetic variations in heat tolerance for fine fescue, and the heterozygosity of SNPs in such two positions could serve as molecular markers in marker-assisted breeding of fine fescue for improving heat tolerance.

Sucrose synthase catalyzes the reversible conversion between sucrose and fructose and NDP-glucose [45]. Sucrose synthase is also part of sugar sensing and signaling transduction pathways, which lead to the regulation of glycolysis [46]. In the current study, SS1 transcript levels were less downregulated in a heat-tolerant cultivar (‘Radar’) than in a heat-sensitive cultivar (‘Longfellow’) of chewings fescue under heat stress. Cultivars with heterozygous alleles of SNP at position 740 of SS1 also had significantly higher Fv/Fm ratio (photochemical efficiency) under heat stress. Our results suggested that heat tolerance in fine fescue could be due to the better maintenance of sucrose synthesis, which could be the result of less interrupted photochemical efficiency. The SNP in SS1 could be a potential candidate gene marker to select for heat tolerance in fine fescue cultivars.

Antioxidant system is an important defense frontline for a plant to cope with abiotic stress, including heat stress [47]. Increases of antioxidant gene expressions and antioxidant protein contents were reported to positively correlate with heat tolerance in several turfgrass species [2,6,15,16,17,48,49]. In this study, heat-tolerant cultivar (‘Radar’) had significantly higher transcript levels of CATA than heat-sensitive counterpart (‘Longfellow’) of chewings fescue under heat stress, showing its positive role in improving heat tolerance at the transcript level. Cultivars with heterozygous alleles of SNPs at position 344 and position 644 in CATA sequence had significantly higher chlorophyll contents than those with homozygous alleles, and cultivars with heterozygous alleles of SNPs at position 344 and 392 in CATA sequence also had significantly lower EL than those with homozygous alleles. It is therefore suggested that more active antioxidant system positively correlated with fine fescue heat tolerance, leading to better cell membrane stability (lower EL) and chlorophyll content. The SNP in CATA could be a potential candidate gene marker to select for enhanced antioxidant systems in heat tolerance in fine fescue cultivars.

Heat shock proteins (HSPs) have been largely reported as protein chaperons for its folding, assembly, translocation and degradation [50], which is strongly induced by abiotic stresses, such as heat [51]. In this study, the transcript levels of HSP90 were more upregulated in heat-tolerant cultivar (‘Reliant IV’) than in heat-sensitive cultivar (‘Predator’) of hard fescue, indicating its positive correlation with heat stress response and protection. Cultivars with heterozygous alleles at position 282 of HSP90 also had significantly higher photochemical efficiency than those with homozygous alleles. The heterozygosity of SNP in position 282 and its correlation with photochemical efficiency suggested the importance of HSP90 in protecting heat damages in photochemical systems for maintenance of heat tolerance for fine fescue cultivars, and the utilization of this marker to select for heat-tolerant germplasm.

Actin, in the form of actin microfilament bundles, is of great importance in plant cellular functions, such as cell growth, cytoplasmic streaming, cell transport, and signaling [52,53,54]. The actin cytoskeleton has been also demonstrated to connect plasma membrane, and was influenced by membrane fluidity [55,56]. In this study, ACTIN expression levels were more induced in heat-tolerant cultivars (‘Radar’ and ‘Reliant IV’) than in heat-sensitive cultivars (‘Longfellow’ and ‘Predator’) of both hard fescue and chewings fescue. Cultivars with homozygous alleles of SNP at position 731 of ACTIN had significantly lower EL and higher chlorophyll content than those with heterozygous alleles. Greater upregulation of ACTIN gene expression in the heat-tolerant cultivars than those in the heat-sensitive cultivars indicated the involvement of plant growth in heat tolerance of fine fescue, which could lead to higher chlorophyll content and less damage (EL). The significant marker correlation of ACTIN with both membrane stability and chlorophyll content suggested its potential utility in marker-assisted breeding, or genetic modification for improving heat tolerance in fine fescue.

In summary, expressions of candidate genes in several major metabolic pathways and stress responses were also positively correlated with heat tolerance manifested by the genetic variations in leaf chlorophyll content, photochemical efficiency, and membrane stability in fine fescue cultivars. SNP markers were developed for genes involved in photosynthesis (RuBisCO activase, Photosystem II CP47 reaction center protein), carbohydrate metabolism (Sucrose synthase), energy production (ATP synthase), growth regulation (Actin), oxidative response (Catalase), and stress protection (Heat shock protein 90). Significant differences of plant physiological parameters existed between cultivars, based on heterozygosity of these SNP markers, indicating their validity of predicting heat tolerance. The development of SNP markers for candidate genes in heat tolerance may allow marker-assisted breeding for the development of new heat-tolerant cultivars in fine fescue.

## Figures and Tables

**Table 1:** Primers used for qRT-PCR of selected genes.

Gene	Accession	Primer Sequence	
RBCL	GO796655	Forward	GTTCAGTTCAGTCAAGTGGTAG
		Reverse	GGAGTTCAGATCAGGCAAAG
RBCS	GO852616	Forward	GATACTACGACGGCAGGTA
		Reverse	CATAGGCGTCAGGGTACT
RCAB	GO876625	Forward	GGCTATCTTGAAGTGGGTAAAT
		Reverse	GTTGGCTTTGGAGGGATAAA
CP47	GO847696	Forward	CAGTAGTATCGCTGCTGTATTT
		Reverse	AGTATCCTGATCCCACTGATAA
CAB	GO884317	Forward	TGAGGCTGTCTGGTTCAA
		Reverse	ACCCATGAGCACAACCT
FNR	GO803921	Forward	GACTTCGCTGTGAGCAG
		Reverse	GTTGTCCTTCTTGAGCATTTC
ATPA	GO855374	Forward	GCTTCCCTGAATACCCAAAG
		Reverse	CTTTCAACCCTTTCAACCAATC
ATPASE	GO869342	Forward	ACACTGTGCCTTGCTTAC
		Reverse	GGGCGAACAGGATCTTTAATA
GAPDH	GO789276	Forward	CCGCACTGAACATCGTG
		Reverse	TGATAACGAGGTCCACCA
SS1	GO874751	Forward	CATCAAAGGACAAGGAGGAG
		Reverse	CATTACGGACACGGTTCAT
ACTIN	GO839904	Forward	TGTTCTCAGTGGAGGTTCTA
		Reverse	CCTTTCAGGTGGTGCAATA
TUBB	GO795142	Forward	CATGTAAGGAGATCCGTGTG
		Reverse	CCTAGTGGTATTGCGTTGATAA
HSP26	DT690825	Forward	GACGACAAGGAGGTGAAGA
		Reverse	GGATGACGAGCGTGTAGT
HSP70	GO882325	Forward	ACAAGATCACCATCACCAAC
		Reverse	CTTGGCATCAACCTTCTTCT
HSP90	GO882272	Forward	ACTACGTCACCAGGATGAA
		Reverse	TGACCTCATAGCCCTTCTT
CATA	GO877704	Forward	TGATGGATGGCTGGATCT
		Reverse	GATCTAGGCATGGTCTCTTATTG
EF1A	HO060093	Forward	CAGATCAATGAGCCCAAGAG
		Reverse	ATTCACACCAGTCTCAACAC

**Table 2:** Summary of reference length, sequencing length, number of total SNPs found, and estimated SNP frequency in candidate genes.

Gene	Reference Length (bp)	Sequencing Length (bp)	Total SNP Number (Q > 10)	Frequency (bp per SNP)
*ACTIN*	1004	600	58	10.3
*ATPA*	1036	1008	9	112.0
*CATA*	1081	569	53	10.7
*CP47*	1041	631	10	63.1
*HSP90*	1056	924	58	15.9
*RCAB*	1210	361	11	32.8
*SS1*	1193	491	17	28.9

**Table 3:** Primers used for sequencing of candidate genes.

Gene	Accession	Primer Sequence	
*ACTIN*	GO839904	Forward	GTGCTTGACTCTGGTGATG
		Reverse	AGTGCTAAGAGAGGCCAA
*ATPA*	GO855374	Forward	CGTGGTCAACGAGAACTTAT
		Reverse	GCATCAAGTTCCATCTTTCTTT
*CATA*	GO877704	Forward	CGACGAGGAGGACAAGTT
		Reverse	TGAGCTCATGGCTGACC
*CP47*	GO847696	Forward	CTGGCTCGATGGCTTTATAC
		Reverse	GCAGCGATACTACTGGAAAG
*HSP90*	GO882272	Forward	TGACTGGGAGGAGCATTT
		Reverse	TAGTCTCTGCTGGACATGTAG
*RCAB*	GO876625	Forward	TTGATGAACTTGGCGGATAA
		Reverse	GATAACCCATCCTTCGTATACAT
*SS1*	GO874751	Forward	CATGTCCATCTACTTCCCATAC
		Reverse	GCGATAGAGCTCAGCATTAC

## Supplementary Materials

Supplementary materials can be found at www.mdpi.com/1422-0067/19/1/116/s1.
